# Different Efficacy of Burosumab on Physical Performance and Serum Phosphate in Adult Patients with X-Linked Hyphophosphatemic Rickets during the First Six-Month of Treatment

**DOI:** 10.3390/jcm12082906

**Published:** 2023-04-17

**Authors:** Teresa Arcidiacono, Nadia E. Foligno, Elena Brioni, Arianna Bologna, Giovanna Weber, Stefano Mora, Marco Pitea, Corrado Vitale, Giuseppe Vezzoli

**Affiliations:** 1 Nephrology and Dialysis Unit, IRCCS San Raffaele Scientific Institute, 20132 Milan, Italy; arcidiacono.teresa@hsr.it (T.A.); foligno.nadiaedvige@hsr.it (N.E.F.);; 2 Postgraduate School of Nephrology, Vita Salute San Raffaele University, 20132 Milan, Italy; 3 Department of Pediatrics, IRCCS San Raffaele Scientific Institute, Vita Salute San Raffaele University, 20132 Milan, Italy; 4 Laboratory of Pediatric Endocrinology, IRCCS San Raffaele Scientific Institute, 20132 Milan, Italy; 5 Nephrology and Dialysis Unit, Mauriziano Hospital, 10128 Turin, Italy

**Keywords:** X-linked hypophosphatemic rickets, burosumab, hyphophosphatemia, FGF23

## Abstract

Burosumab is a monoclonal anti-FGF23 antibody used to treat patients with X-linked hypophosphatemic rickets (XLH). Its effect on serum phosphate and physical performance was compared in patients during a 6-month treatment with burosumab. Eight adult patients with XHL were treated with burosumab (1 mg/kg s.c. every 28 days). In the first 6 months of treatment, calcium-phosphate metabolism variables were measured, and muscle performance (tested with chair and walking test) and quality of life (tested with fatigue, BPI-pain and BPI-life questionnaires) were estimated. A significant increase in serum phosphate was observed during the treatment. From the 16th week, serum phosphate became significantly lower than its value in the 4th week. No patients had serum phosphate below the normal range at the 10th week, but seven patients were hypophosphatemic in the 20th and 24th week. All patients improved the execution time of the chair test and walking test, which reached a plateau after the 12th week. BPI-pain and BPI-life scores significantly decreased from baseline to the 24th week. In conclusion, a six-month burosumab treatment may significantly improve the general condition and physical performance of adult patients with XLH; this improvement was more stable and more indicative of treatment efficacy than that of serum phosphate.

## 1. Introduction

X-linked hypophosphatemic rickets (XLH) is a rare monogenic disorder caused by loss-of-function mutations at the *PHEX* gene (Xp22.11), which encodes for the zinc-dependent phosphate-regulating endopeptidase homolog, X-linked (PHEX) [1]. *PHEX* mutations may impair the hydrolytic inactivation of fibroblast growth factor 23 (FGF23), the hormone secreted by osteocytes with a key role in phosphate homeostasis and XLH pathogenesis [2]. FGF23 downregulates the expression of phosphate reabsorption carriers NPT2 in the apical membrane and the activity of the 1a-hydroxylase of the 25-hydroxyvitamin D (25(OH)D) in proximal tubular cells. Thus, the increase in FGF23 serum concentration in XLH patients leads to the inhibition of tubular reabsorption and intestinal absorption of phosphate, the decrease in serum phosphate concentrations, and the development of osteomalacia and rickets. Patients with XLH may have limb deformities such as coxa vara and genu valgum or varum, and disproportionate short stature with shorter limbs since their first infancy; they may also have craniosynostosis with possible neurologic defects, fractures, pseudo-fractures and dental abnormalities, such as enamel defect, abscesses, enlarged pulp chambers and periodontitis [3]. The early administration of the conventional treatment with oral phosphate supplements and calcitriol may heal rickets, support growth and limit dental disorders [4]. If not treated or surgically corrected, bone deformities cause severe disability in adult patients associated with long-term complications, such as osteoarthritis, enthesopathies, spinal stenosis, pseudo-fractures, bone pain and hearing loss [3,5].

In addition to these alterations, patients with XLH may develop a myopathy that is more severe in adults and is characterized by decreased skeletal muscle density and peak force and muscle pain. Myopathy may especially affect lower limb muscles because of bone and joint deformities, but it is not confined to these body regions. Its causes remain unclear, although it could result from the reduced availability of phosphate and 1,25-dihydroxyvitamin D (1,25(OH)_2_D), potentially leading to an alteration of energy and mitochondrial metabolism, whereas the possible direct involvement of FGF23 in XLH myopathy remains controversial [6,7,8]. Response of myopathy to therapy of XLH also remains unclear.

Recently, burosumab, a human monoclonal antibody inactivating FGF23, became available for the treatment of XLH; compared to the conventional therapy (calcitriol and phosphate salts), the pharmacological activity of burosumab can specifically counteract FGF23 activities and the pathogenetic mechanism of XLH [9,10,11]. In Italy, burosumab is currently indicated in children with severe bone involvement, whereas it can only be used as a compassionate treatment in adult patients. The efficacy of burosumab treatment may be estimated through both the increase in serum phosphate and the improvement in physical performance in adult patients [3]. Therefore, the present work compared the efficacy of burosumab in improving physical performance and serum phosphate in a small cohort of adult patients with XHL treated for six months in a compassionate treatment program.

## 2. Materials and Methods

Response to burosumab was studied in eight patients with XLH followed in the Nephrology and Mineral Metabolism outpatient clinics of San Raffaele Hospital in Milan (n = 7, patients 1–7) and Mauriziano Hospital in Turin (n = 1, patient #8). All patients were adults (aged between 20 and 59 years, Table 1) and had diagnosis of XLH in their infancy. Genetic analysis documented *PHEX* gene mutations in all patients. Patients #5 and 6 were brothers. Patients were treated with conventional therapy (calcitriol and phosphate salts) during their infancy and adolescence, but they were not adherent to pediatrician prescriptions, and their treatment was discontinuous.

Patients with XLH were selected for burosumab treatment due to their disability. All patients had short body height (Table 1) and reported bone and muscle pain, particularly in lower limbs, in the morning after waking up. They also reported low resistance to physical effort, weakness and inability and low quality of life. Only patients with these disturbances were selected for burosumab therapy, which was proposed to patients as a compassionate treatment. The clinical history of all patients included multiple orthopedic operations to correct limb deformities during childhood. Patient #3 underwent surgical correction of spinal canal stenosis at the age of 43 years, 3 years before starting burosumab, and was treated with calcitriol for a short time after this surgical procedure. Patient #6 had severe scoliosis. All patients were followed by pediatric departments in different hospitals during their childhood and youth, but they reported incomplete adherence to conventional therapy. Before burosumab therapy, one patient (#4) was taking calcitriol and phosphate salts, one patient (#5) was taking paricalcitol and cinacalcet because of autonomous hyperparathyroidism; the remaining patients were not taking conventional medications (calcitriol and phosphate salts) for XLH for at least one year. No patients suffered from other diseases in addition to XLH or were taking drugs for reasons other than XLH. No patients had low blood levels of hemoglobin.

All patients had short body height (Table 1) and reported bone and muscle pain, particularly in lower limbs, in the morning after waking up. They also reported low resistance to physical effort, weakness and inability and low quality of life. Because of these disturbances, burosumab was proposed to patients as a compassionate treatment. The treatment program was activated in each patient after approval of the manufacturer (Kyowa Kirin) and the Institutional Ethical Committees of San Raffaele and Mauriziano Hospitals. Informed consent was obtained from all subjects involved in this study. Burosumab was supplied by the manufacturer and stored in the Hospital Pharmacy before being administered to patients. Patients suspended conventional therapy seven days before the beginning of burosumab treatment, if in progress. Burosumab was injected subcutaneously in the nephrology outpatient clinic by a nurse at the dose of 1 mg/kg body weight every 4 weeks (28 days ± 2 days). All patients received six burosumab injections to be considered in the present study.

Blood samples were collected at baseline (immediately before the first burosumab injection) and before each burosumab injection to measure serum concentrations of calcium, phosphate, intact parathyroid hormone (PTH) (Electro-chemiluminescent immunoassay [ECLIA], Roche Diagnostics, Mannheim, German), 1,25-dihydroxycolecalciferol (1,25(OH)_2_D) (ECLIA, Diasorin, Stillwater, MN, USA), 25-hydroxycolecalciferol (25(OH)D) (ECLIA, Roche Diagnostics, Mannheim, Germany), creatinine, bone alkaline phosphatase (bALP) (chemiluminescent immunoassay, Diasorin, Stillwater, MN, USA) and C-terminal telopeptide of type I collagen (CTX) (ECLIA, Roche Diagnostics, Mannheim, German). Serum phosphate was also measured 2 weeks after each burosumab injection. Intact FGF23 was measured at baseline in all patients except in patient #8 using a two-step ELISA method (Diasorin, Stillwater, MN, USA). In three patients, FGF23 was also measured after 6 months of treatment.

Physical ability was investigated in patients with five time sit-to-stand chair test, 10 m walking test and fatigue questionnaire performed at baseline and before each burosumab injection. Patients also responded to BPI-pain and BPI-life questionnaires, investigating pain and life quality at the same time points [12,13,14,15,16]. Chair rise test was executed with patients sitting on a chair with feet on the floor slightly back from the knees and arms crossed and held to the chest; the test consisted of standing 5 times and returning to sitting on the chair after each time stood; the time to complete this test was measured with a chronometer and was normal for values less than 14 s. [13]. Walking test measured the time taken by the patient to walk 10 m on a flat path at the fastest possible speed [14]. Fatigue and BPI questionnaires were filled by 7 patients during the follow-up as patient #7 was not compliant in filling them in. 

Additionally, the excretion of calcium and phosphate was measured in 24 h urine; tubular reabsorption of phosphate (TRP) and tubular threshold for phosphate reabsorption (TmP/GFR) were estimated at the baseline and after 4, 12 and 24 weeks of treatment [17] in 7 patients, as patient #7 was not compliant in collecting urine.

Patients underwent multifrequency bioelectrical impedance analysis (BIA) using the impedentiometer BIA101 BIVA PRO (Akern, Pontassieve-Florence) and the software Body Gram Plus (Akern, Pontassieve-Florence) to assess skeletal muscle index (SMI) at baseline and after 24 weeks of therapy with burosumab [18].

### Statistical Analysis

Quantitative variables were reported as mean ± SD [range]. Differences between values of quantitative variables detected at baseline and during the follow-up were compared with Wilcoxon test for paired data. Distribution of patients in groups was evaluated with Fisher exact test. Linear Pearson correlations between variables were studied. Statistical analysis was two-tailed and was conducted at α = 0.05 level. It was performed using the SPSS statistical package (IBM, Armonk, NY, USA).

## 3. Results

### 3.1. Baseline Characteristics 

Patient baseline characteristics are shown in Table 1. All patients had hypophosphatemia with low TmP/GFR. Execution time of chair and walking tests was normal in two and five patients, respectively. Scores of fatigue, BPI-pain and BPI-life questionnaires were normal in all patients (n = 7). Serum 1,25(OH)_2_D was in the normal range in all patients but inappropriate to maintain serum phosphate values in the normal range. Patient #8 was taking cinacalcet due to autonomous hyperparathyroidism and had normal serum calcium. Another three patients (#2, 5 and 6) had mild hyperparathyroidism with normal serum calcium; these patients and patient #8 had serum CTX above the normal range. FGF23 was high in six patients, whereas in patient #2, it was inappropriately normal in relation to serum phosphate. 

### 3.2. Serum Phosphate and TmP/GFR during Treatment with Burosumab

**Figure 1:**
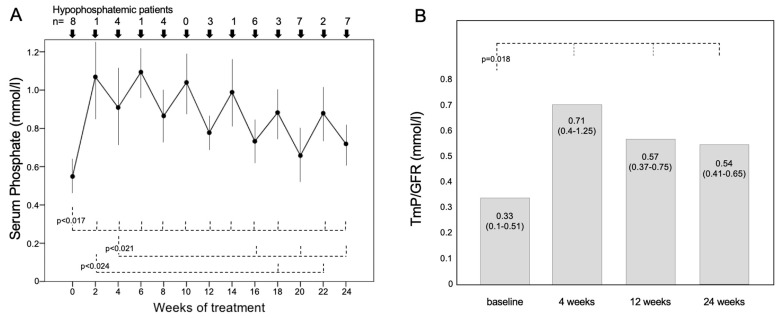
(**A**) Values of phosphatemia during 24 weeks of treatment with burosumab in 8 patients with XLH. Variables are reported as mean ± SD. The drug was injected subcutaneously (1 mg/kg body weight), and serum phosphate was measured two and four weeks after each burosumab injection. The number of patients with serum phosphate below the normal range is reported in the upper part of the figure. (**B**) Values of TmP/GFR measured in 7 XHL patients at the baseline and after 4, 16 and 24 weeks of treatment with burosumab.

Serum phosphate significantly increased during the 6 months of burosumab treatment (Figure 1A): its value was 0.55 ± 0.13 [0.4–0.79] mmol/L at baseline and raised to 1.07 ± 0.22 [0.74–1.4] mmol/L two weeks after the first burosumab injection (*p* = 0.012), and to 0.91 ± 0.24 [0.71–1.41] mmol/L (*p* = 0.012) in the fourth week. After the 4th week, serum phosphate progressively decreased; it remained higher than baseline (at the 24th week, it was 0.72 ± 0.09 [0.65–0.82] mmol/L; *p* = 0.017), but after the 16th week, its concentrations became significantly lower than those at the 2nd or 4th week (Figure 1A).

Serum phosphate was within the normal range (>0.8 mmol/L) in all patients only at the 10th week, whereas it was again below the normal range in four (50%) patients at the 4th and 8th week (*p* = 0.038 vs. baseline) and in three patients at the 12th week of treatment (*p* = 0.013). In the 20th and 24th weeks, seven patients had low serum phosphate (88%; *p* = 0.5 vs. baseline) (Figure 1A). 

TmP/GFR, measured in seven patients, was significantly higher after 4, 12 and 24 weeks of treatment compared with baseline (Figure 1B). 

### 3.3. Physical Tests and Quality of Life Surveys during Treatment with Burosumab

**Figure 2:**
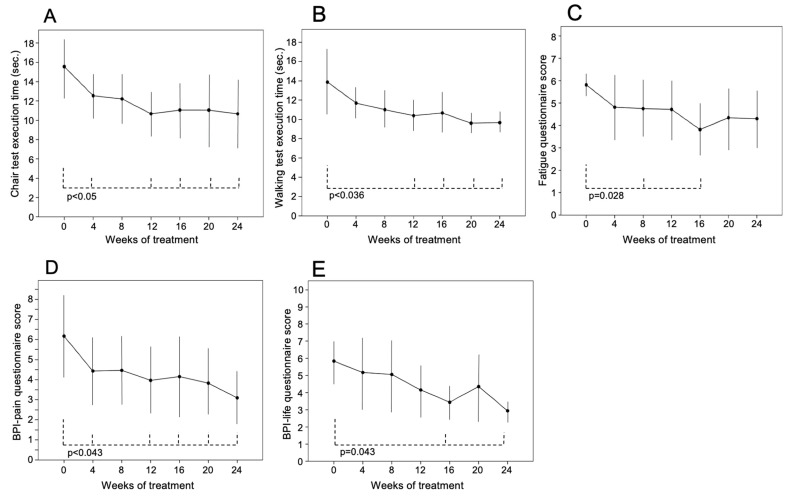
Findings of (**A**) chair and (**B**) walking tests and (**C**) fatigue, (**D**) BPI-pain and (**E**) BPI-life questionnaires during 24 weeks of treatment with burosumab in 8 patients with XLH. Variables are reported as mean ± SD. The drug was injected subcutaneously (1 mg/kg body weight). Physical tests were performed in 8 patients; fatigue, BPI-pain and BPI-life questionnaires were performed in 7 patients.

Patients significantly improved the time to execute physical performance tests and the score of quality-of-life tests during the burosumab treatment (Figure 1). The chair test (16 ± 3 [13–19] s at baseline) and walking test (14 ± 4 [8–12] s at baseline) showed a progressive improvement during the follow-up, and values of both tests became significantly lower than those at the baseline from the 12th week of treatment (chair test 11 ± 3 [8–16], *p* = 0.017; walking test 10 ± 2 [8–13], *p* = 0.036) (Figure 2A,B, respectively). After 12 weeks, the execution time of both tests reached a plateau and was significantly faster than baseline up to the 24th week (chair test 11 ± 4 [8–19], *p* = 0.025; walking test 10 ± 1 [8–12], *p* = 0.017 vs. baseline) (Figure 2A,B, respectively). Chair test was normal for two patients (38%) at the baseline but for seven (88%) in the 24th week (*p* = 0.02), because a patient had severe deformities of the legs and column that hindered normal movement. 

The fatigue questionnaire scores (5.8 ± 0.4 [5.4–6.3] at baseline) significantly improved at the 8th (4.5 ± 1.2 [2.5–6], *p* = 0.028) and 16th week of treatment (3.7 ± 1.3 [2.1–6], *p* = 0.028) and remained substantially stable after the 16th week (Figure 2C). 

The BPI-pain questionnaire scores (6 ± 1.9 [2.5–8] at baseline) significantly decreased during the follow-up. In the 24th week, it was 3.1 ± 1.3 [1.1–5] (*p* = 0.043 vs. baseline values) (Figure 2D). 

The BPI-life questionnaire (5.8 ± 1.3 [4–7.3] at baseline) showed a reduction in its score that was significantly lower than baseline at the 16th (3.4 ± 0.8 [1.6–5.7]; *p* = 0.043) and 24th (2.9 ± 0.6 [1–5.2]; *p* = 0.043) week of treatment (Figure 2E). 

During treatment with burosumab, no significant modification of SMI was detected (9.2 ± 1.3 [7.3–10.6] at baseline vs. 8.5 ± 1.5 [6–10] after 24 weeks of treatment; *p* = 0.3). 

**Figure 3:**
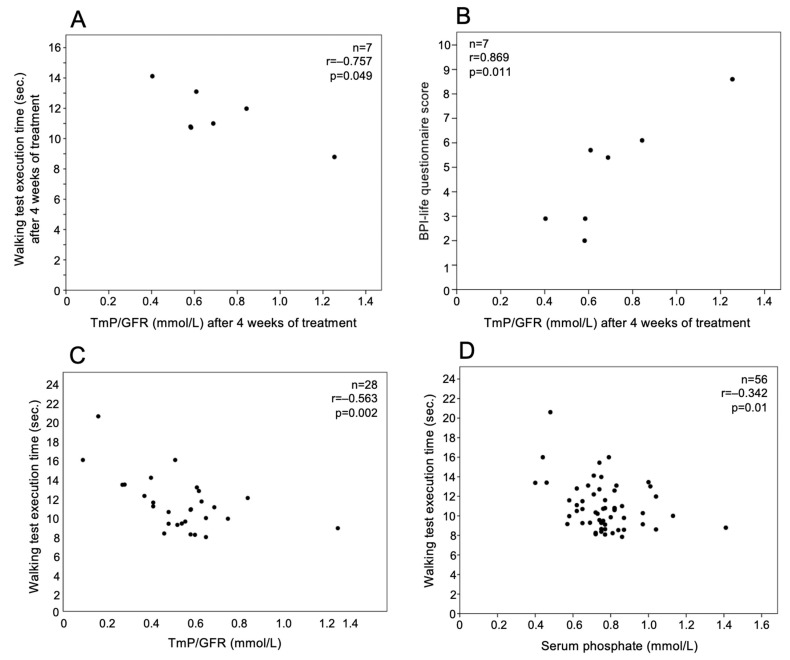
TmP/GFR time was correlated with (**A**) walking test execution time and (**B**) BPI-life score at the 4th week of burosumab treatment in 7 patients with XLH. Considering together all the determinations at different times, walking test execution time was correlated with (**C**) TmP/GFR and (**D**) serum phosphate.

TmP/GFR values detected at the fourth week of treatment were negatively correlated with walking test execution time (r = −0.757, *p* = 0.049) (Figure 3A) and positively with BPI-life questionnaire score (r = 0.869, *p* = 0.011) (Figure 3B) determined at the same time point. Serum phosphate was positively correlated with the BPI-life questionnaire score in the fourth week of treatment (r = 0.839, *p* = 0.018). Considering together all the determinations at different times, the walking test was negatively correlated with serum phosphate (n = 56, r = −0.342, *p* = 0.01) and TmP/GFR (n = 28, r = −0.563, *p* = 0.002) (Figure 3C,D). 

### 3.4. Other Variables of Calcium-Phosphate Metabolisms during Treatment with Burosumab

**Figure 4:**
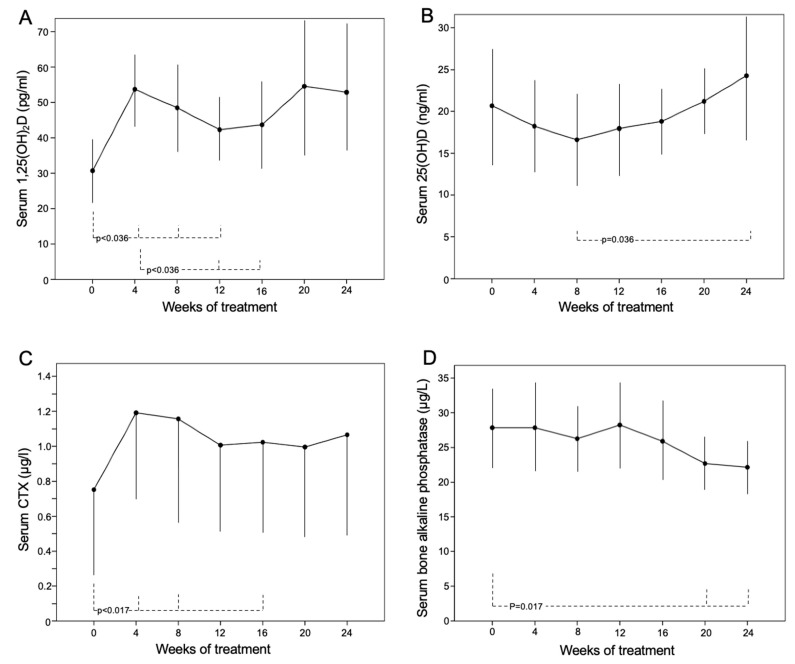
Serum values of (**A**) 1,25(OH)_2_D, (**B**) 25(OH)D, (**C**) CTX and (**D**) bALP during 24 weeks of treatment with burosumab (1 mg/kg body weight every 28 days) in 8 patients with XLH.

Serum 1,25(OH)_2_D significantly increased from the 4th week (54 ± 11 [33–72] vs. 31 ± 9 [26–51] pg/mL at baseline; *p* = 0.017) to the 12th week of treatment (42 ± 9 [30–69] pg/mL; *p* = 0.036 vs. baseline). Its concentration in the 12th and 16th weeks was lower than that in the 4th week (Figure 4A). The serum concentration of 25(OH)D slightly increased in the 24th week related to baseline, as patients were supplemented with cholecalciferol (Figure 4B). PTH remained unchanged, whereas an increase in CTX (Figure 4C) and a reduction in bALP were also observed during the treatment (Figure 4D).

Serum FGF23 was also measured at the end of the follow-up in three patients. Its values were increased by 253%, 385% and 369% in patients #1, 2 and 5, respectively. Values of hemoglobin remained normal during the treatment.

Values of SMI did not change after 24 weeks of treatment with burosumab compared to baseline values (8.5 ± 1.5 [6–10] vs. 9.1 ± 1.3 [7.3–10.7], respectively; *p* = 0.31).

## 4. Discussion

In the present study, the effect of burosumab on serum phosphate and physical performance was evaluated in adult patients with XLH to identify which one was more indicative of its therapeutical efficacy [8,19,20]. Burosumab is a monoclonal antibody inactivating FGF23 that restores tubular phosphate reabsorption, serum phosphate and 1,25(OH)_2_D synthesis in patients with XLH. These patients suffered from muscle and articular discomfort and disability and had significant limitations in their personal and social lives. Thus, burosumab may also improve skeletal muscle disability, physical performance and quality of life in XLH patients, in addition to bone remodeling [2,10,21]. This may be attributed to the larger availability of phosphate and 1,25(OH)_2_D, but also to the loss of the negative effect of FGF23 on skeletal muscle cells and osteoblasts [8,22,23]. Curiously, quality-of-life questionnaires showed normal scores in a large portion of our patients, probably because they were used to managing their disabilities and underestimating them. Findings in our patients showed that scores of physical performance and quality of life progressively improved during the first weeks of treatment with burosumab and reached stable levels after 12–16 weeks, as was previously observed in children [24,25]. In our patients, burosumab also confirmed its already known positive effect on phosphate metabolism, 1,25(OH)_2_D synthesis and bone remodeling; however, burosumab decreased its effect on phosphate metabolism during the follow-up as serum phosphate concentrations significantly dropped after an early serum peak and again became lower than the normal range in most of the patients after 16 weeks of treatment. A similar trend was previously observed in children in a phase 3 clinical trial [24]. These findings suggest that burosumab decreases its therapeutical efficacy on serum phosphate after its first injections, whereas it may maintain its efficacy on physical activity and quality of life during the follow-up. The decline of its effect on serum phosphate could be attributed to counterregulatory mechanisms, as the response of serum phosphate to burosumab may support FGF23 secretion by osteocytes that may take serum phosphate back to lower concentrations during the treatment. This leads to a new balance between phosphate and FGF23, as suggested by our findings in three patients and in a previous work, although burosumab interfered negatively with FGF23 determination [26]. In addition, the development of antibodies against burosumab could take part in these mechanisms, even though this event has not been reported so far. Other factors, such as iron deficiency, are less likely due to the normal hemoglobin blood levels during the treatment.

The improvement in skeletal muscle performance induced by burosumab was not explained by a variation in skeletal muscle mass or detected by BIA, but may result from a better function of skeletal muscle cells [27] sustained by the recovery of serum phosphate and vitamin D that became more available for metabolic processes [6,7] and by the inactivation of the hypothetical inhibitory effect of FGF23 on skeletal myocytes [23]. Skeletal myocytes were found to express the receptor for FGF23 that, thus, may directly influence myocyte activity. In vitro experiments observed that FGF23 induced premature senescence in mesenchymal stem cells derived from skeletal muscle through an oxidative-stress pathway [23,28]. As an alternative, FGF23 might influence skeletal muscle activity through a decrease in myocyte phosphate content impairing skeletal muscle function [7,29,30]. 

The different response of serum phosphate and physical tests to burosumab during the six months of follow-up suggests that physical tests and quality-of-life surveys may be more indicative of burosumab efficacy than serum phosphate in XLH patients. Therefore, physical recovery may be considered an important target of burosumab treatment, and findings of physical performance tests could be considered the most useful markers of the clinical efficacy of this therapy in adult patients with XLH. Tests investigating skeletal muscle performance could be included in the clinical follow-up for an exhaustive evaluation of patients with XLH. The walking test appeared to be more indicative of burosumab’s effect, as its findings were correlated with serum phosphate concentrations and tubular phosphate reabsorption in our patients. 

Limitations of our study are the small sample size, as we enrolled only eight patients from two hospitals, and the compassionate use of burosumab, for which the established dosage (1 mg/kg bw) could not be changed. Moreover, physical parameters were measured four weeks after each burosumab injection, whereas the more marked effect of burosumab on serum phosphate was recorded 2 weeks after the drug injection, and patients reported a better physical and working ability in the first two weeks after the burosumab injection than during the following two weeks. These observations indicate that the dosage of burosumab or the interval between injections could be revised to optimize its clinical effects in single patients. A patient (#8 in Table 1) had autonomous hyperparathyroidism and was treated with cinacalcet in addition to burosumab; this patient showed a more marked tendency to decrease its serum phosphate below the normal range after burosumab injections.

In conclusion, treatment with burosumab improved the general condition and physical performance of patients, although its effect on serum phosphate may decline after the first weeks of treatment. Tests investigating skeletal muscle performance, pain, and disability may be included in the clinical routine to evaluate patients with XLH and may help in driving the treatment with burosumab in these patients.

## Figures and Tables

**Table 1:** Baseline characteristics of patients with XHR after suspension of calcitriol and phosphate salts for 7 days. The reference range of the variables is in brackets. PTH: intact parathyroid hormone; bALP: bone alkaline phosphatase; CTX: C-terminal telopeptide of type I collagen; FGF23: fibroblast growth factor 23; 25(OH)D: 25-hydroxyvitamin D; 1,25(OH)2D: 1,25-dihydroxyvitamin D; TRP: tubular reabsorption of phosphate; TmP/GFR: tubular threshold for phosphate reabsorption; BPI: brief pain inventory. BPI: brief pain inventory.

Sex		Age (Years)	Body Weight (kg)	Height (m)	BMI (kg/m^2^)	Hip/Body Height Ratio	Chair Test (s)	Walking Test (s)	Serum Phosphate (mmol/L)	Serum Calcium (mmol/L)	PTH (pg/mL)	bALP (µg/L)	CTX (ng/L)	FGF23 (pg/mL)	25(OH)D (ng/mL)	1,25(OH)_2_D (pg/mL)	Urine Calcium (mmol/24 h)	TRP (%)	TmP/GFR (mmol/L)	Fatigue Test	BPI- Pain	BPI- Life
Normal Range						(=0.5)	(<14)	(<16)	(0.8–1.5)	(2.1–2.54)	(15–65)	(5–26)	(<580)	(10–50)	(>20)	(20–79)	(2–7.5)	(>75)	(0.9–1.35)	(<7)	(<10)	(<10)
1	M	31	59	1.52	25.5	0.49	19	16	0.44	2.39	55	23	410	102	15	26	2.86	38	0.18	5.8	2.5	6.6
2	F	47	57	1.5	25.3	0.47	14	11	0.62	2.31	75	19	583	35	19	34	3.86	77	0.48	7	7.5	10
3	M	46	77	1.55	31.9	0.5	19	16	0.79	2.3	56	12	230	67	14	51	2.2	77	0.61	6.3	7	7.3
4	F	20	54	1.38	28.4	0.5	15	13	0.4	2.48	43	22	554	72	27	30	1.64	68	0.27	5.4	5.3	5.3
5	M	31	77	1.6	30.2	0.48	13	11	0.62	2.3	70	26	778	238	35	29	3.1	67	0.41	5.2	8	6.4
6	M	34	77	1.46	35.9	0.47	17	13	0.46	2.41	90	55	1270	159	17	27	4.56	62	0.3	5.8	5	5.9
7	M	40	85	1.61	32.8	0.48	11	10	0.58	2.48	54	15	388	159	13	20	4.45	66	0.38	-	-	-
8	F	59	60	1.35	32.7	0.44	18	21	0.48	2.38	257	52	1803	-	25	29	0.84	34	0.18	6.1	7	4
mv-		39	68	1.5	30	0.48	16	14	0.55	2.38	88	28	752	119	21	31	2.94	43	0.33	5.8	6	5.8

## Data Availability

The data presented in this study are openly available in the San Raffaele Open Research Data Repository.
